# Adaptive control for downhole nuclear magnetic resonance excitation

**DOI:** 10.1038/s41598-023-31031-x

**Published:** 2023-03-14

**Authors:** Guanghui Shi, Lizhi Xiao, Sihui Luo, Guangzhi Liao, Yan Zhang, Xiang Zhang, Jian Zhong, Wanli Zhu, Xueli Hou

**Affiliations:** 1 College of Geophysics, China University of Petroleum, Beijing, 102249 China; 2 College of Carbon Neutral Energy, China University of Petroleum, Beijing, 102249 China; 3 Huanding Energy Services, Beijing, China; 4 China National Logging Corporation, Xi’an, China

**Keywords:** Geophysics, Techniques and instrumentation

## Abstract

Nuclear magnetic resonance (NMR) measurements are performed with the pulse sequence and acquisition parameters set by the operator, which cannot be adjusted in real time according to sample characteristics. In one acquisition cycle, usually thousands of high-power pulses are transmitted and thousands of echo points are acquired. The power consumption cause the RF amplifier to overheat, and large amounts of acquired data may be invalid. Therefore, the optimization of excitation and acquisition processes is necessary to improve measurement efficiency. We explore a scheme for the real-time measurement of the samples by adaptively regulating the pulse sequence, which adapts the variable *TE* pulse sequence as the reconnaissance mode. The appropriate pulse sequence and reasonable parameters (*NE*, *TE*) can be selected according to the relaxation characteristics of the samples.This adaptive control strategy has great significance in guiding both dynamic and static measurements, and it is especially suitable for occasions where low magnetic field gradients and diffusion terms can be ignored. We also design a test circuit for adaptive control, which has the function of automatic parameter adjustment. By adjusting parameters such as the number of refocusing pulses, echo spacing, etc., the effective measurement of the samples can be achieved in practice.

## Introduction

NMR technology is an important means used to analyze the properties of the fluids filling the pore space in the rocks, and the quantitative identification of hydrocarbons in these fluid is beneficial to the exploration and production of oil and gas^1–3^. In the past 10 years, nuclear magnetic technology has made great progress in both hardware and data processing methods. Downhole NMR instruments mainly focus on how to improve logging efficiency and the richness of pore fluid information, while benchtop NMR core analyzers focus more on how to improve the resolution of measurements. However, downhole and benchtop instruments still have some common problems, such as signal-to-noise ratio (SNR), power consumption of the power amplifier, acquisition time. These problems are directly related to the measurement mode of NMR instruments. Different measurement modes have different pulse sequence parameters. Pulse sequence parameters include echo spacing (*TE*), wait time (*TW*), number of echoes (*NE*), pulse width, etc^4^. NMR logs featuring multiple Carr-Purcell-Meiboom-Gill (CPMG) sequences with varying wait time and echo spacing were collected to obtain inverted longitudinal relaxation time (*T*_1_) or transverse relaxation time (*T*_2_) data. For one of the pulse sequences, its parameters remain unchanged during the measurement.

Using the same pulse sequence parameters in oil-barren strata as those used in hydrocarbon-rich strata will result in loss of transmission energy and excessive collection of invalid information, which will affect the stability of the tool, logging time, and logging accuracy. Many experts have done some research work on optimization of NMR pulse sequences. In 2012, M. Ronczkad et al. explored an acquisition method of variable echo spacing based on CPMG pulse sequence^5^. This scheme reduces the number of pulses and energy consumption, but it does not have the function of real-time adjustment and the pulse sequence still works with the preset parameters. In 2019, Yiqiao Song et al. proposed a class of real-time optimization methods that conducts stochastic analyses on the acquired data and in turn updates and optimizes the subsequent measurements^6^. This method provides direction on the optimization of acquisition parameters. In terms of hardware real-time parameter adjustment, David Ariando et al. designed a portable adaptive intelligent data acquisition system and proposed an intelligent data acquisition method for measuring the *T*_*2*_. This method uses the objective function of the current sample for closed-loop control. Once converged, the most suitable *TW*、*TE* and *NE* can be determined^7^. This scheme requires multiple inversion, which prolongs the data processing time and reduces the real-time performance in regulation. In 2020, Yiqiao Tang et al. used a supervised learning model to learn relevant sensor signals to classify samples, and implemented a closed-loop analysis method on a microprocessor to measure the relaxation properties of complex fluids in real time^8^. The disadvantage of this scheme is that the adjustment of pulse sequence parameters is limited to a limited range. In the field of MRI,there are numerous case studies of adaptive measurement that restore a high fidelity image from partially observed measurements^9^. In 2022, Inbal Beracha et al.demonstrated that an adaptive pulse sequence for measuring the transverse relaxation time of metabolites in-vivo improves upon the precision of static approaches by a factor of ≈1.7- or, alternatively, accelerates acquisiton 2.5-fold^10^. The aforementioned schemes for changing the pulse sequence parameters improve the accuracy of sample evaluation to a certain extent, but there is currently no scheme for optimizing the pulse sequence in the downhole environment considering the tool’s motion state.

In a stationary measurement,tool response is governed by sample and fluid parameters.We can keep adjusting the pulse sequence parameters until we get a satisfactory result. In case of tool motion, however, the measurement physics gets complicated^11^. The typical wireline logging speed is 150 m/h, while the acquisition of a single echo train may take anywhere from a few to tens of seconds (including the time for polarization)^12,13^. In the logging process, the logging object is constantly changing because the tool is in motion. We can simulate downhole NMR measurements in the laboratory by changing the test samples during the measurement.

According to the real-time measurement results, the pulse sequence parameters can be adjusted in time to meet the needs of sample testing with different characteristics. This adaptive adjustment method is similar to the feedforward control in the control system. The main purpose is to realize adaptive NMR measurement under the condition of sample change and overcome the problem with conventional methods,i.e., pulse sequence parameters cannot be automatically adjusted.

## Measurement basics

NMR logging focuses on the measurement of the relaxation and diffusion properties of formations. NMR spectrometers analyze a formation’s relaxation properties by transmitting a sequence of pulses while acquiring decaying spin echo trains^14,15^. For practical applications, it is essential to use multi-pulse sequences that generate echoes. In particular, the activation that consist of the CPMG sequence and related sequences forms the basis of most NMR logging measurements. The sequence consists of an initial 90° excitation pulse, followed by a long train of identical refocusing pulses that are phase-shifted by 90° relative to the excitation pulse^16^. Echoes are formed between the refocusing pulses, their width is about the duration of a refocusing pulse, the echoes take on a stable shape and the amplitude decays with the relaxation rate^17,18^. Some activation parameters can be changed by the field engineer—for example, number of echoes (*NE*), wait time or polarization time (*TW*), echo spacing (*TE*), running average (*RA*), and number of operating frequecncies (*XF*)^1,19^. Because the relaxation times of the formations are distributed over a wide range, ranging from milliseconds to seconds, it is important for reasonable parameter setting.

Considering a classic CPMG sequence sampled with echo spacing *TE*, an echo is created between successive 180° pulses so that the signal amplitude at the *n*th echo is equal to the expression^20^

1 $$E(nTE) = \sum\limits_{j = 1}^{NF} {F_{j} (1 - e^{{{{ - TW} \mathord{\left/ {\vphantom {{ - TW} {T_{1j} }}} \right. } {T_{1j} }}}} )} e^{{ - \frac{nTE}{{T_{2j} }}}} , \quad (n = 1,2, \ldots NE)$$

Here, *TW* is the wait time that lets the fluid protons repolarize between successive CPMG sequences, *NF* is the number of fluid phases present in the pore space. For each fluid* j* present in the porous rock, *F*_*j*_ is its fractional volume or porosity, *T*_*1j*_ is its longitudinal relaxation time, *T*_*2j*_ is its transversal relaxation time.

**Figure 1:**
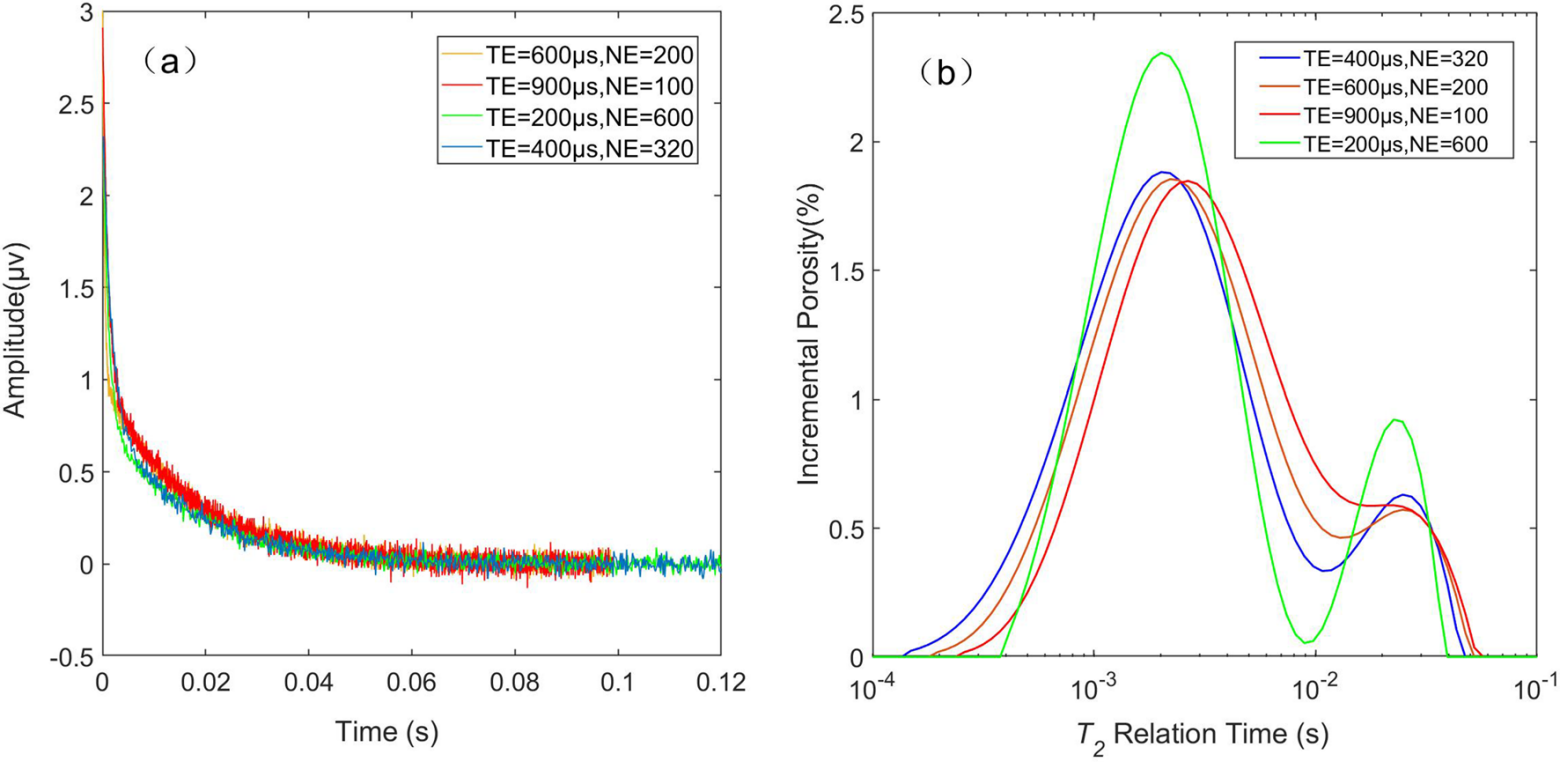
Original echo trains and *T*_2_ spectra of the same sample with different pulse sequence parameters, assuming that *TW* is sufficiently large. (**a**) Echo trains data points acquired with different parameters. (**b**)* T*_2_ spectra obtained by the inversion of echo trains with different acquisition parameters.

To avoid the effect of incomplete polarization, *TW* should be equal to or greater than 3 or 5 times the longitudinal relaxation time (*T*_1_). The role of *TE* is mainly manifested in the measurement of diffusion relaxation. In the slow relaxation component of large pores and weak magnetic field gradients, when *TE* is small, the diffusion effect is small or even negligible. Using formula (1), the echoes are collected and inverted with different measurement parameters, and the signal strength of each *T*_2_ component can be obtained. As shown in Fig. 1, it can be seen that the performance of the same sample on the *T*_2_ spectra varies considerably.

For a spectrometer, it is necessary to collect effective information as much as possible through the pulse sequence and truly reflect the attenuation trend of the echo train. The number of scans can be set to filter random noise to varying degrees. The setting of the number of echoes determines the acquisition time^21^. The setting of the wait time affects the degree of polarization of the fluid in the pores. Due to the hardware limitation of the instrument, the echo spacing cannot be set to excessively small values^22^. The smaller the echo spacing, the more obvious the ringing effect of the antenna, and the lower the signal-to-noise ratio of the probe signal^23,24^. In order to capture the complete relaxation decay, it is necessary to use the shortest echo spacing to acquire the short relaxation component, but the long relaxation component requires a larger number of echoes. *T*_*2*_ ranges from approximately 0.5 ms to 500 ms in clastics. However, much longer transverse relaxation times have been found in carbonates.

## Adaptive control scheme

**Figure 2:**
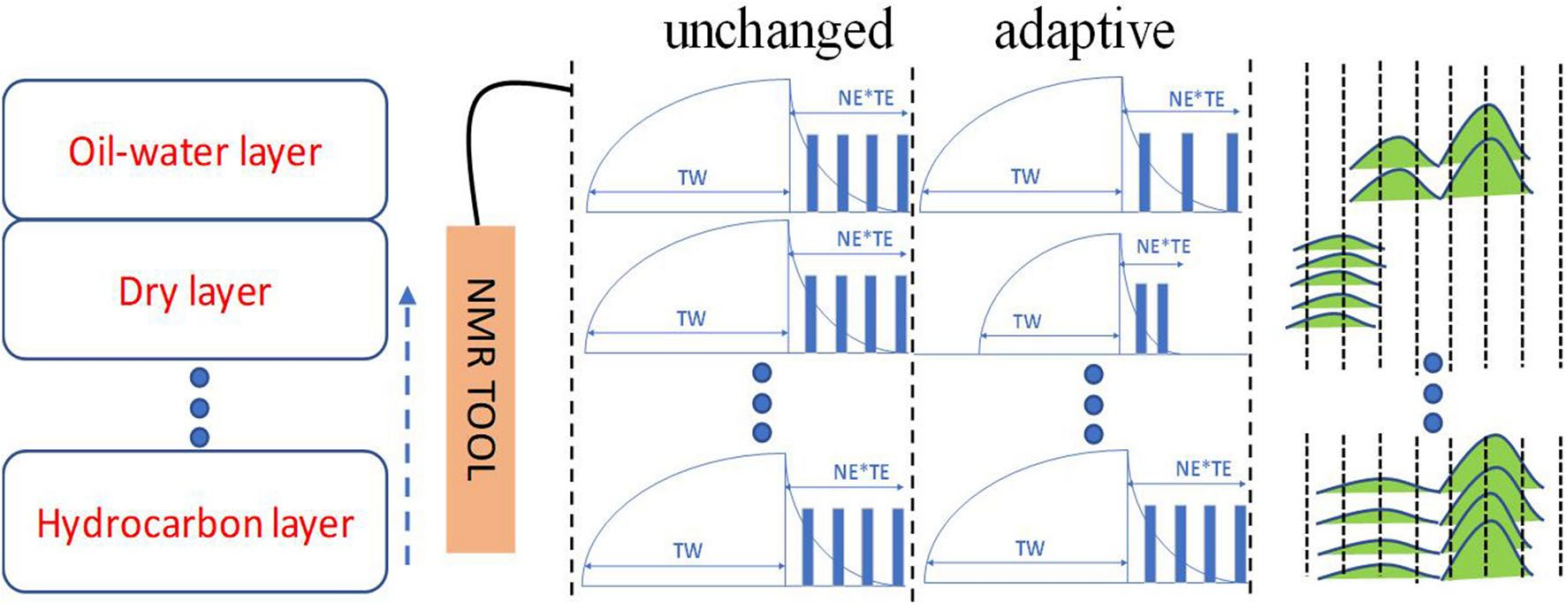
NMR logging tool for formation classification based on T2 spectra^33,34^. The design goal is to achieve real-time adjustment of pulse sequence parameters in different layers while the parameters in the conventional method remain unchanged.

In the process of downhole motion measurement, NMR instruments will encounter rock formations with different pore structures and different permeability characteristics^25^. Some layers are rich in oil and gas, while others are not. As shown in Fig. 2, we have roughly divided the measurement well interval into several types of layers, including oil-bearing layer, oil–water layer, dry layer, water layer and other layers. The oil-bearing layer has oil shows, and the *T*_2_ spectra for this layer show obvious oil characteristics, which need to be monitored. The dry layer with very little oil and gas content is generally a rock layer with low porosity and low permeability^26^, and its *T*_2_ spectra barely shows any characteristics of oil and gas. The water layer is the layer dominated by formation water, which can be easily identified by conventional instruments^27–29^.

When there is no human intervention, the pulse sequence parameters will remain unchanged. However, it is unnecessary to collect echo information with the same parameters from different layers. In view of the fact that the logging object is in motion, its characteristics are difficult to predict. Therefore, it is highly necessary to conduct research on the automatic optimization of pulse sequence parameters taking into account sample changes. Compared with the previous measurement methods with preset parameters, the proposed measurement method can collect sample information in real time, which plays an important role in adjusting the pulse sequence parameters and thereby achieving closed-loop control.

### A. Key factors to the realization of a closed-loop control system

**Figure 3:**
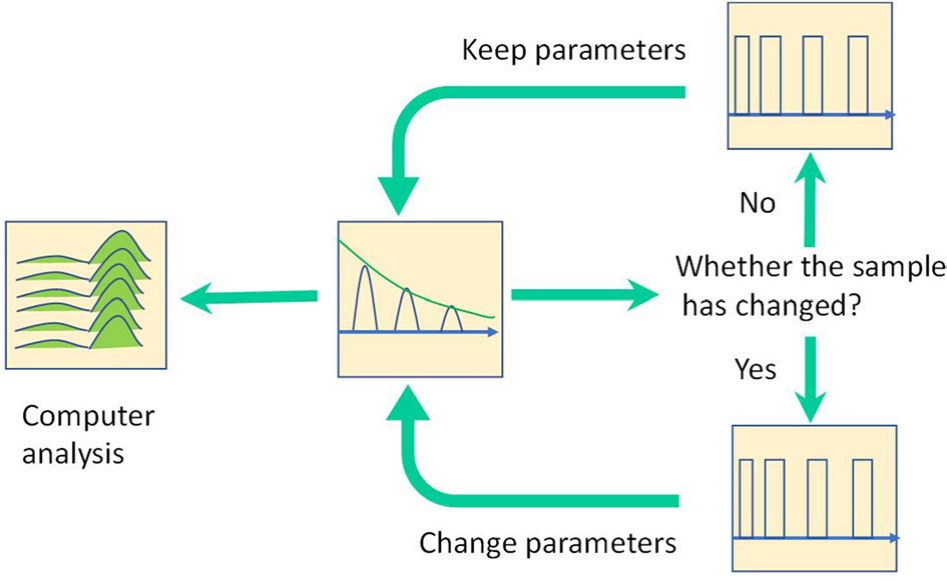
A closed-loop control scheme for samples in motion.

The classic adaptive control system is mainly used in industrial control systems to solve the problem of system deviation caused by external interference and maintain the desired state in time by adjusting the parameters. The objects in industrial control systems are indeterminate and unknown. This is the same for the adaptive control system proposed in this paper. The difference is that the object considered in this paper is in a state of motion relative to the NMR measuring instrument. The basic idea of the control system is shown in Fig. 3. The spectrometer control circuit should adjust the parameters in time based on the results of real-time data processing.

The key factors for achieving closed-loop control are as follows:

1. The collected data used as the basis for parameter adjustment should be capable of effectively reflecting all *T*_2_ components in the formation samples.
2. Given the fact that the sample is in motion, the hysteresis caused by parameter adjustment should be reduced, and real-time data processing should be ensured as far as practicable.

### B. Pulse sequence program optimization

At present, *T*_1_ mode and *T*_2_ mode are still the basic modes of NMR logging. *T*_2_ mode can sample all components in a single measurement, while *T*_1_ measurement requires data generated by multiple sets of echo sequences^30–32^. This paper takes porosity logging in *T*_2_ mode as an example to study the adaptive control strategy.

When the spectrometer detects changes in the sample, it quickly switches to the reconnaissance mode, and the relaxation properties of the sample are acquired by the reconnaissance sequence. Here, a variable *TE* pulse sequence is introduced as the reconnaissance sequence^5^. Being different from the classic CPMG sequence,the echo spacing of the variable *TE* pulse sequence increase linearly.

The *TE* of the variable *TE* pulse sequence can be given by:

2 $$TE_{{\text{n}}} { = }TE_{\min } { + }TE_{\min } {*(}N{\text{E}}{ - }1)*\frac{1}{FM}$$

where *FM* is the echo spacing increase coefficient. Starting from the minimum echo spacing *TE*_*min*,_ the echo spacing is increased by 1/*FM* each time according to the arithmetic progression.

**Figure 4:**
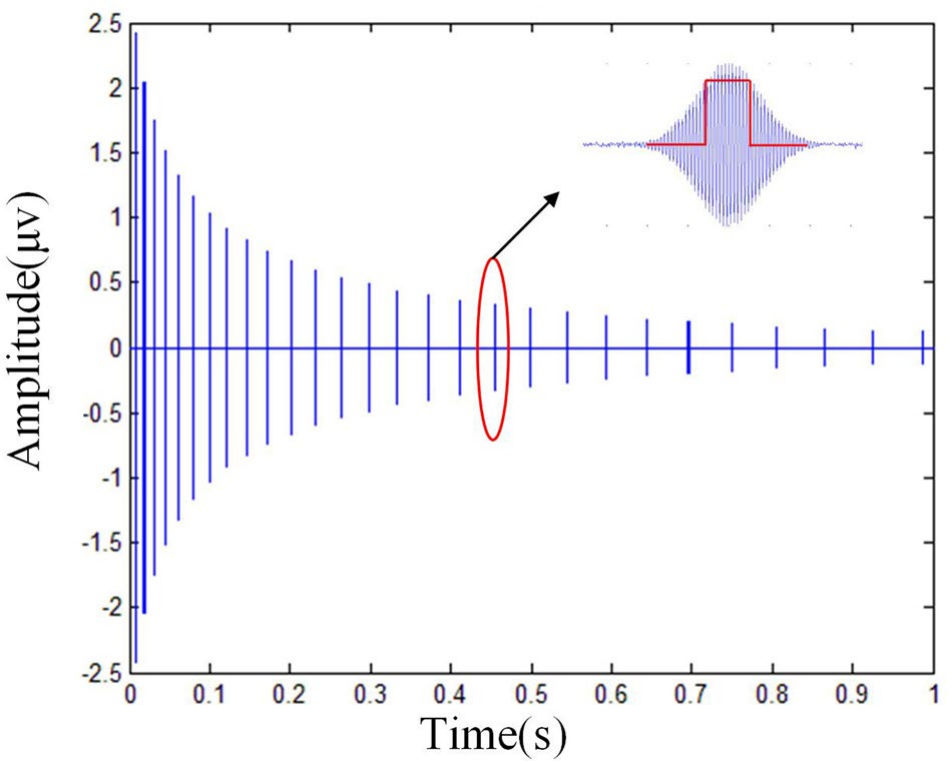
Echo acquisition using the variable *TE* pulse sequence.Each blue line represents the echo that appears after the 180° pulse, and the echo width is the same as the 180° pulse width, but only 32 echo cycles (about 64 μs) are acquired by opening the acquisition window, echo amplitude is considered to be uniform in this window.

Figure 4 is the echo train generated by the sequence. Compared with the CPMG sequence, the advantage of this sequence is that fewer echo points are collected at the same acquisition time, which can reduce data transmission and processing time.

The variable *TE* pulse sequence can avoid the loss of long-relaxation components and accurately obtain short-relaxation component data. Within the allowable range of diffusion errors, the information collected by the variable *TE* pulse sequence can still be used for formation evaluation,so we use it as the reconnaissance mode. The normal CPMG is used as an evaluation mode to provide more accurate information for sample measurements.

The approximate distribution ranges of *T*_*2*_ and *T*_*2max*_ can be obtained through the inversion algorithm from the data collected by the reconnaissance sequence, and then the echo spacing and the number of echoes of the evaluation sequence can be adjusted. The adjustment of the echo spacing helps improve the resolution of relaxation measurements. The system chooses the parameters based on the desired type of pulse sequence (CPMG or Variable *TE* sequence). The adjustments of the number of echoes and the echo spacing satisfy the following relationship:

3 $$\begin{gathered} NE*TE \ge 3*T{}_{2\max }, \, (CPMG) \hfill \\ \sum\nolimits_{1}^{n} {TE_{{\text{n}}} } \ge 3*T{}_{2\max }, \, (Variable{\text{ TE}}) \hfill \\ \end{gathered}$$

In addition, we can estimate *TW* based on empirical formula:

4 $$TW > k*3T_{2\max }$$

Usually *k* is approximately equal to 1.5. If the *T*_*2*_ spectrum shows the presence of gas, the value of *k* is larger.

After the data acquisition and processing platform obtains the optimal parameters, the sequence switching command and the optimal parameters are sent to the main control unit, and the new acquisition parameters are quickly executed after an acquisition cycle.

Throughout the whole process of well interval measurement, effective reservoirs account for a small proportion, and most of the layers may be dry layers. In order to improve the logging efficiency, the variable *TE* pulse sequence can be maintained in dry layers, and the pulse sequence can be switched to the CPMG sequence in hydrocarbon-bearing layers, and the pulse sequence parameters can be adjusted appropriately.

### C. Judgment index of parameter adjustment

**Figure 5:**
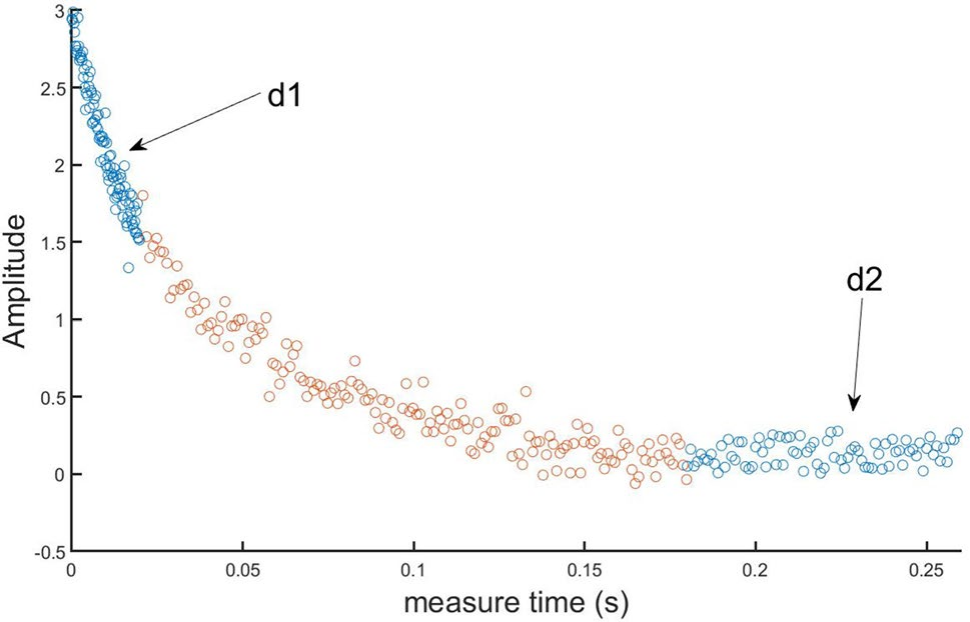
Scatter plot of the echo train. The circles represent echo data points acquired during a pulse sequence, and the blue circles represent the echo amplitudes of the head and end of the echo train.

We need to judge, from the collected data and information, whether the sample has changed. Figure 5 shows the original echo train obtained by collecting a series of echo points. We compute the sum of the head and end of the echo train, which are denoted by *d*_1_ and *d*_2_, respectively^7^. Their mean $\overline{d }$ and standard deviation ${\sigma }_{d}$ are computed by collecting eight separate data points of *d*_1_ and *d*_2_^7^. The values of *d*_1_ and *d*_2_ measured for the next pulse sequence dataset are then compared with their statistics from the previous eight scans. The condition for determining whether the sample has not changed is as follows:

5 $$\left|{d}_{1}^{{\prime}}-\overline{{d }_{1}}\right|\le 3{\sigma }_{{d}_{1} } \,\, \mathrm{and} \,\, \left|{d}_{2}^{{\prime}}-\overline{{d }_{2}}\right|\le 3{\sigma }_{{d}_{2}}$$

where ${d}_{1}^{{\prime}}$ and ${d}_{2}^{{\prime}}$ are the newly collected data points, ${\sigma }_{{d}_{1}}$ and ${\sigma }_{{d}_{2}}$ are the standard deviations of *d*_*1*_ and *d*_*2*_, and $\overline{{d }_{1}}$ and $\overline{{d }_{2}}$ are their means. We believe that, if Eq. (5) is satisfied, the sample has not changed, and the system will continue to execute the current pulse sequence parameters and monitor whether the current sample has changed. Similarly, we believe that, if the data fails to satisfy Eq. (5), the sample has changed, and the system will switch the pulse sequence to the variable *TE* pulse sequence and re-optimize the pulse sequence parameters for the new sample to be detected.

## Experiments

### A. Hardware system

The NMR instruments consists of a transmit/receive (TX/RX) controller for generating or coupling electromagnetic signals.The TX/RX controller directs the pulsed magnetic field source to output a specific pulse sequence, and detects the NMR spin echo phenomenon associated with the pulse sequence. The pulse sequence generator is the core component of the TX/RX controller. Pulse sequence generators adjust pulse sequence by changing the pulse duration, phase, amplitude, shape, and time between each pulse. Therefore, the pulse sequence generator can be programmed to provide suitable parameters such as *TE*, *TW* and *NE*. In addition, the transmission timing cooperates with some necessary modules such as reception, isolation, and discharge modules, to realize the excitation and acquisition of echo signals.

**Figure 6:**
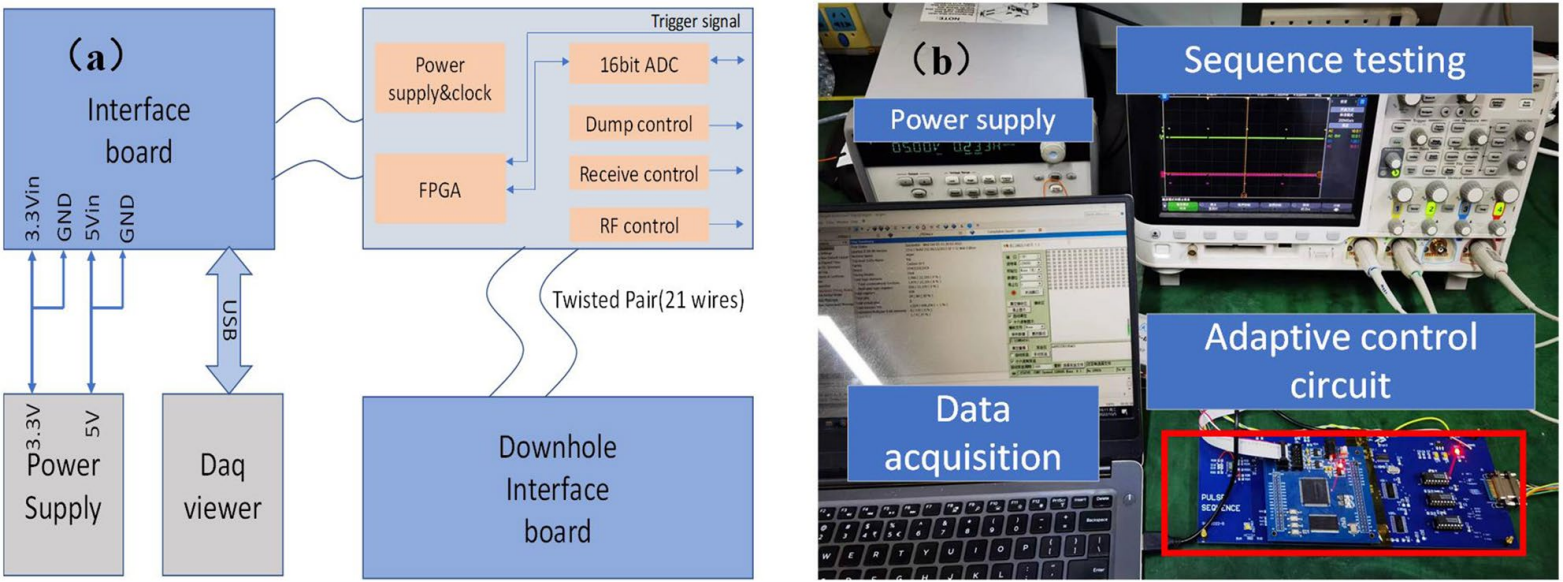
Design and testing of circuit system. (**a**) Block diagram of hardware platform; (**b**) Testing of adaptive control circuit.

Figure 6 shows the frame drawing of the overall circuit design.The hardware test platform consists of the main control circuit, analog front-end, and data transmission and acquisition platform(DAQ viewer). The model of the A/D acquisition chip is AD9238, the windowing time for single echo acquisition is 50 μs, and the conversion result is sent from the communication interface to the DAQ viewer at a rate of more than 2 Mbps. The pulse sequence switching command and the latest parameters are also sent to the FPGA by the DAQ viewer, and the FPGA executes the latest parameters after one acquisition cycle.

**Figure 7:**
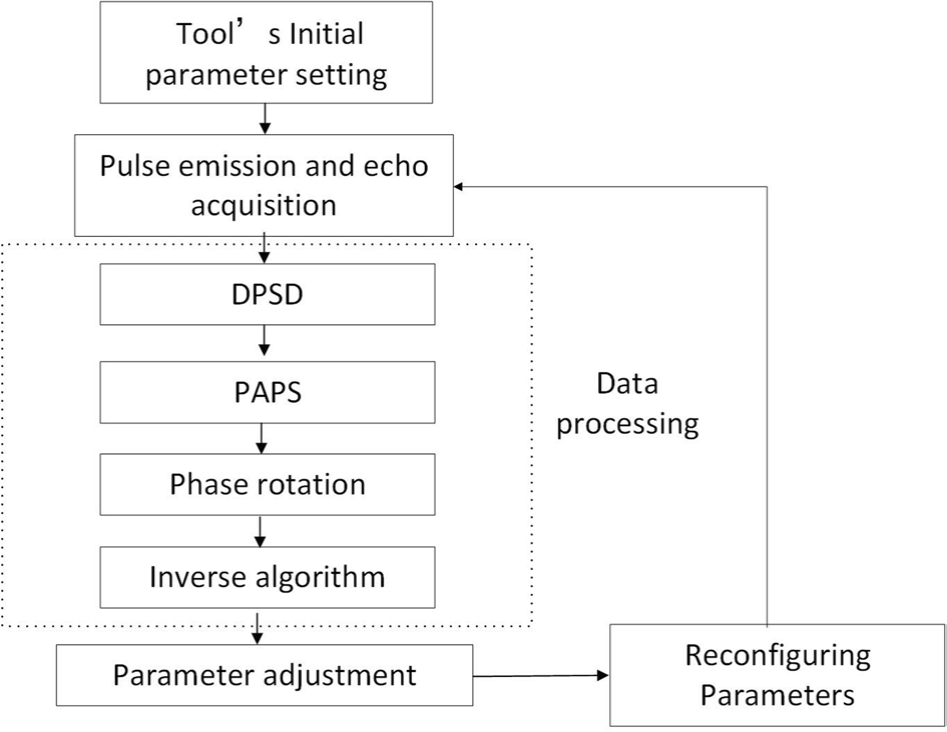
Schematic diagram of the FPGA for adaptive adjustment of the pulse sequence.

In order to achieve the purpose of real-time data acquisition and feedback control, a high-speed data acquisition and processing chip and peripheral circuits based on system-on-chip (SOC) are used. By integrating the Field Programmable Gate Array (FPGA) and Advanced RISC Machine (ARM) processor on one chip, SOC has two architectures to achieve complementary advantages. While FPGA completes high-speed acquisition and calculation, it realizes data transmission with the main processor through the AXI on-chip bus, which provides more favorable support for realizing the real-time performance of the system. The main idea of adaptive adjustment of the pulse sequence is implemented inside the FPGA by means of a state machine, as shown in Fig. 7.

**Figure 8:**
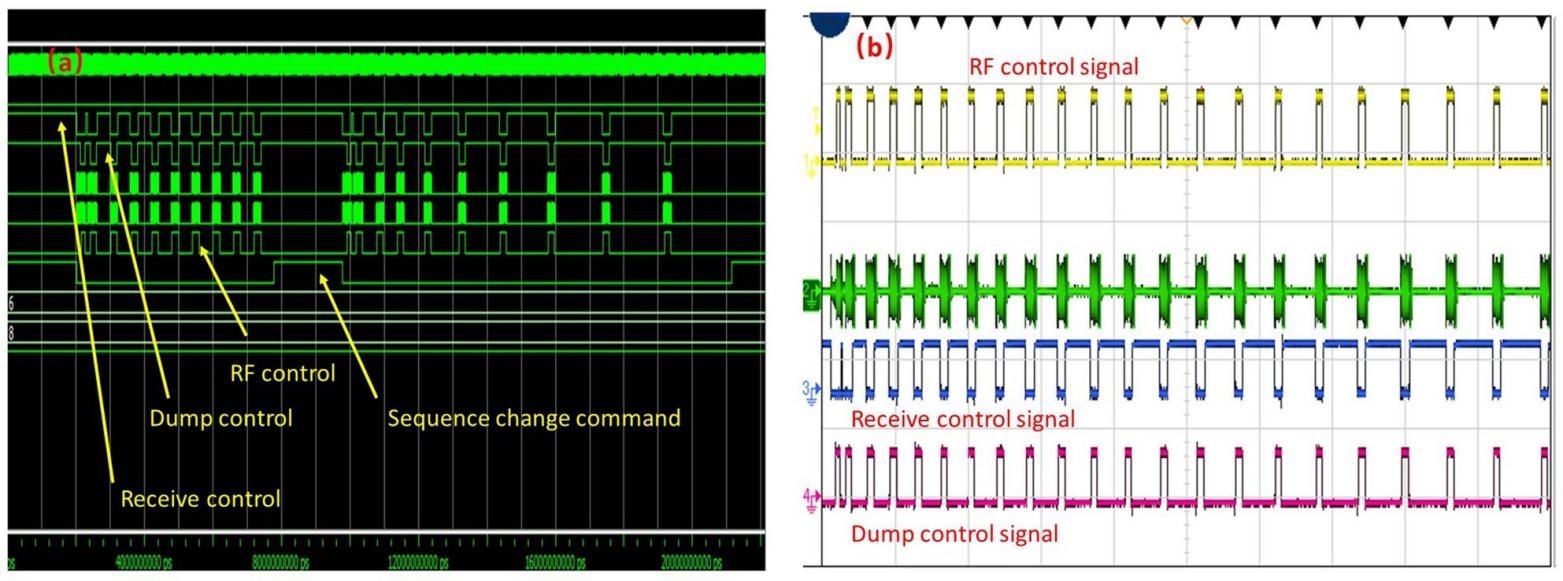
Pulse sequence timing control of nuclear magnetic circuit. (**a**) Pulse sequence dynamic switching simulation results including receive control signal, dump control signal and RF control signal; (**b**) the signal obtained by the oscilloscope test.

The specific data acquisition process is described below. The data acquired by the A/D acquisition chip is first stored in the FIFO, and the data is read from the FIFO and then converted into the AXI-stream data format. This method can speed up data processing and reduce the impact of data on timing. The frequency of the ADC configuration clock is 4.8 MHz, and that of the AXI bus clock is 50 MHz. The clock multiplier can effectively prevent data overflow of the buffer module. The PS terminal obtains 256 data points of each echo and then performs phase-sensitive detection to obtain the real and imaginary parts of the information contained in the signal. For echo acquisition, the SNR should be taken as the goal, and data processing and analysis can be performed only when a sufficient SNR is obtained. To eliminate the effects of noise signals such as ringing, a superposition operation is required. After 2 or 4 sets of PAPS data are collected, the attenuation trend of the echo train can be initially obtained. Figure 8 shows the simulation and debugging results for the circuit.

### B. Continuous measurement experiment

Because many engineering problems are involved, the design scheme presented in this paper does not have the conditions for downhole testing. For this reason, the relevant tests are carried out in the laboratory environment.

**Figure 9:**
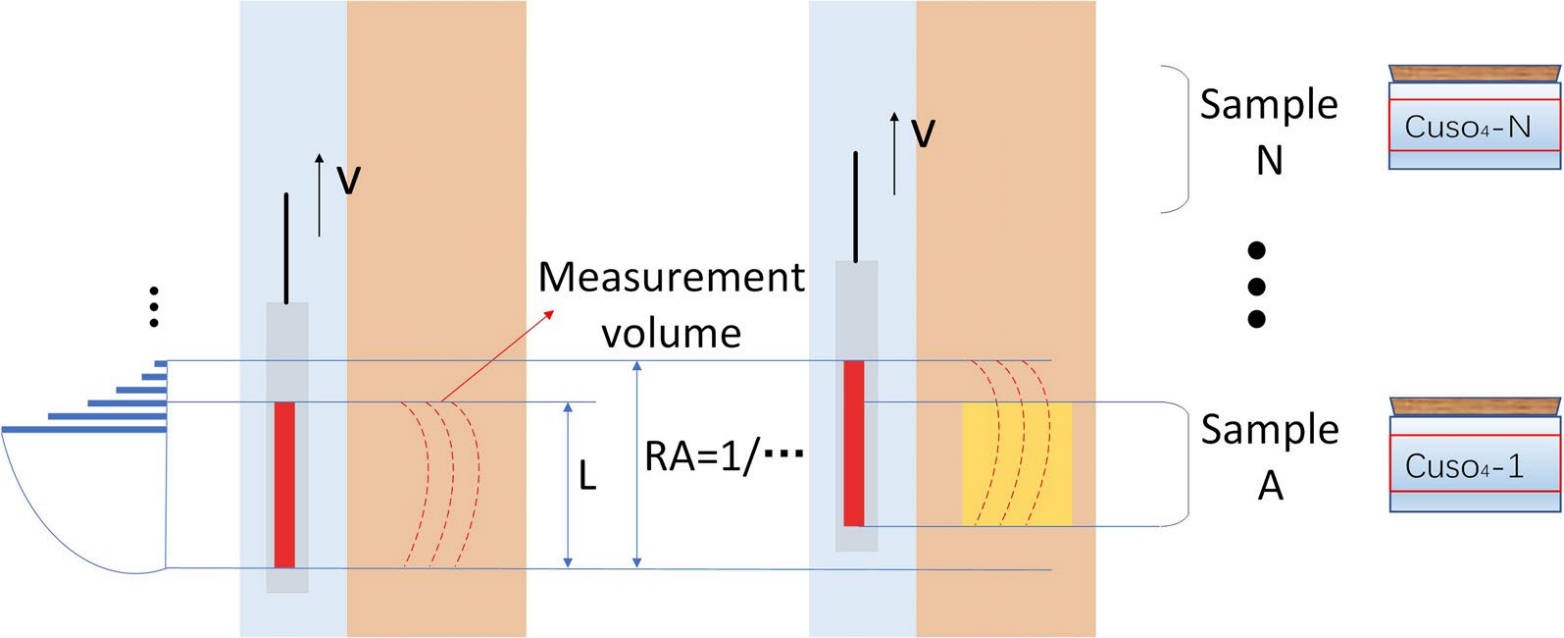
Diagram of an instrument in motion. The logging tool is moving at a speed of v, and the red rectangle represents the RF coil with a length of *L*. The length is about 60 cm. If the tool does not move during the measurement cycle (i.e., a stationary reading is obtained), the vertical resolution (VR) equals the length of the antenna (*L*).

In Fig. 9, The measurement volume is changing slowly when the logging tool moving. The sensitive volume can change 10% during a CPMG sequence. To maintain an acceptable logging speed, a 10% loss of accuracy is typically accepted^1^. The yellow area in the figure is the effective detection area caused by motion, which can be regarded as the first test sample. We can simulate downhole NMR measurement in the laboratory by changing the test samples during the measurement. The samples were sequentially placed into the detection area of the core analyzer with a 1.0'' short-echo-spacing antenna. The model of the probe part is LIME-MRI-D2, the magnetic field strength is 470 GS, the volume of sample solution is 10 mL, and there is no magnetic field gradient. We prepared three samples for continuous measurement, which were cuso_4_ solution samples with different concentrations, and two of them were packed in reagent bottles with separators as one sample, which showed double peaks on the *T*_*2*_ spectra. The *T*_*2max*_ values of the three samples (cuso_4_ solution_1, cuso4 solution__2_, cuso_4_ solution_3) were approximately 10 ms, 100 ms, and 300 ms, respectively. The program design is as follows: the wait time is 6 s, the variable *TE* pulse sequence is used as the initial sequence, the minimum echo spacing is 100 μs, and the number of pulses is 986. After a change in samples is detected, the pulse sequence is adjusted according to the *T*_*2*_ spectrum. When the spectral peak is less than 50 ms, the pulse sequence is switch to the CPMG with *TE* = 60 μs to improve the resolution; when the spectral peak is greater than 200 ms, the echo spacing can be increased appropriately.

**Figure 10:**
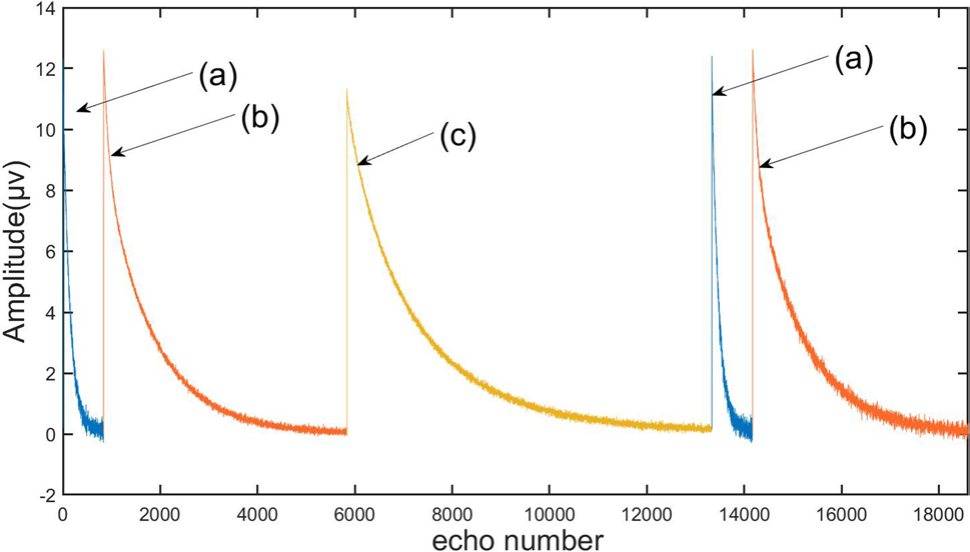
The data points of effective echo trains obtained from three different samples are measured continuously. When testing sample a, the data obtained by the detection sequence determines that the *T*_*2max*_ of the sample is 10 ms through inversion, and the main control unit quickly adjusts the pulse sequence, and the length of the pulse train is reduced from the original value of 2 s to 50 ms. When testing sample b, the sample *T*_*2max*_ was determined by inversion to be 100 ms, and the pulse sequence was adjusted to a CPMG sequence with 5000 echoes. When testing sample c, the pulse sequence remained unchanged due to the larger *T*_*2max*_.

Figure 10 shows the echo trains collected from the three samples circulating into the detection area in turn. From the echo trains, it can be seen that the echo train lengths of different samples are different. If the conventional method is used to test the three samples, there may be oversampling or undersampling. This problem can be avoided by adaptive adjustment. Table 1 shows the parameters used for adaptive measurement of the pulse sequence.

**Table 1:** Comparison of pulse sequence parameters used for continuous measurement of three different samples.

	Sample a	Sample b	Sample c
TW	36–60 ms	360–600 ms	1080–1800 ms
NE	834	5000	7500
TE	60 μs	100 μs	200 μs

The test results show that for dynamic measurements, it is feasible to adjust the pulse sequence according to the relaxation characteristics obtained by the detection sequence. Adaptive control measurements offer several advantages:

1. Using the variable *TE* sequence as the reconnaissance sequence cyclically, the relaxation characteristics of samples with a large range of *T*_*2*_ values can be obtained with less sampling data.
2. The SNR of the adjusted pulse sequence is significantly higher for the same superposition time.
3. The reduction of the number of transmitted pulses can prevent the probe and power amplifier from being overheated.

### C. Discussion of the time-delay problem

The experiment above simulates the real-time optimization of the pulse sequence while the downhole instrument is in motion. Since the parameters are adjusted after observing the change of the sample, there must be a time-delay problem. There are two solutions to the time-delay problem. One solution is to build a forecasting model, and the other solution is to shorten the lag time. The research on forecasting models in NMR logging covers a broad area, which is not considered for the time being, and the instrument detection is allowed to have a certain time delay. The specific analysis is described below.

**Figure 11:**
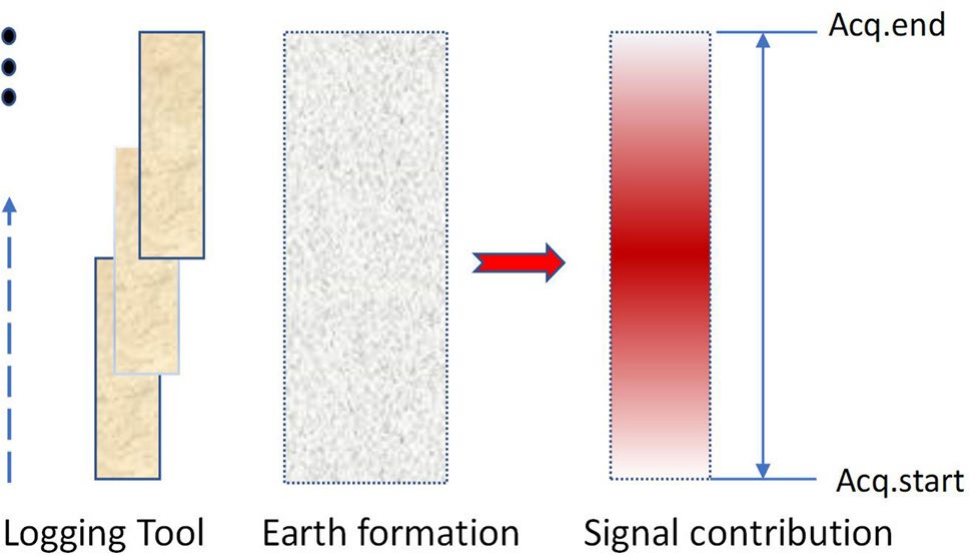
Contribution of the detection area to the acquired signal in one acquisition cycle during the upward movement of the NMR logging tool.

The speed of the upward movement of the logging tool is about 150 m/h. Iin principle, it is required that there is an overlapping area in the detection area of the instrument during the complete pulse sequence. As shown in Fig. 11, in the corresponding detection area from the beginning to the end of the pulse sequence, the contribution of formation samples to the NMR signal first increases and then decreases.

Assuming that the thickness of the overlap region is 0, the region where the red arrow is located contributes the most to the signal because it is always within the detection range. In this worst case, it is feasible to predict the acquisition parameters for the next location using the acquired data for this range because there is a common detection area for both measurements.

For a nuclear magnetic antenna with a length of 60 cm, in order to ensure that there is an overlapping area in the acquisition cycle, the acquisition cycle time is less than 14 s, which is sufficient for a pulse sequence. Moreover, we can even perform multiple rounds of superposition to improve the signal-to-noise ratio of the measurement. To improve the vertical resolution of well logging and mitigate the delay-time problem, it is necessary to minimize the time for detecting the sample, which includes the acquisition time of the echo train, the transmission time, and the data processing time.

## Conclusions

This paper focuses on the method and system design of adaptively adjusting the pulse sequence according to the relaxation properties of samples, and initially achieves the dynamic adjustment of the measurement mode. The adaptive control scheme proposed in this paper has certain significance for guiding both desktop NMR analysis and downhole measurement. Due to the existence of "dry layers" in the well, timely and effective identification and adjustment will help to improve the logging efficiency. In this paper, a controllable variable *TE* pulse sequence is proposed. Using this pulse sequence as the test sequence can effectively reduce the energy consumption and the amount of invalid data without stopping the logging operation. In addition, it can avoid the loss of effective reservoirs.

Wireline logging usually uses multiple frequencies and complex observation modes to obtain petrophysical parameters and fluid information. Although the proposed adaptive control scheme only considers a single frequency, multiple frequencies can still be used in this scheme. The reconnaissance sequence can be set as an auxiliary group in the activation mode to provide guidance for setting the parameter of other groups.

Pulse sequence adaptive adjustment is a closed-loop control strategy for adjusting the pulse sequence parameters according to the original data or inversion results. The adaptive control scheme in this paper does not require an accurate mathematical model, and it is simple and practical. It can be identified according to the collected data and processing results, and can quickly respond to process changes.

In this work, we mainly implement the underlying framework of adaptive master control. There is still some areas to be further improved in the real-time data processing and analysis of the host computer, such as the real-time processing of underground low-noise data. The data inversion results can be used to divide the formation in detail to determine the pulse sequence with the best parameters.

## Data Availability

The datasets generated during and/or analysed during the current study are available from the corresponding author on reasonable request.
